# Separating neural and vascular effects of caffeine using simultaneous EEG–FMRI: Differential effects of caffeine on cognitive and sensorimotor brain responses

**DOI:** 10.1016/j.neuroimage.2012.04.041

**Published:** 2012-08-01

**Authors:** Ana Diukova, Jennifer Ware, Jessica E. Smith, C. John Evans, Kevin Murphy, Peter J. Rogers, Richard G. Wise

**Affiliations:** a Cardiff University Brain Research Imaging Centre, School of Psychology, Cardiff University, Cardiff, UK; b Institute of Psychological Medicine and Clinical Neurosciences, School of Medicine, Cardiff University, Cardiff, UK; c Department of Experimental Psychology, University of Bristol, Bristol, UK

**Keywords:** Simultaneous EEG–FMRI, Caffeine, BOLD, Auditory oddball

## Abstract

The effects of caffeine are mediated through its non-selective antagonistic effects on adenosine A_1_ and A_2A_ adenosine receptors resulting in increased neuronal activity but also vasoconstriction in the brain. Caffeine, therefore, can modify BOLD FMRI signal responses through both its neural and its vascular effects depending on receptor distributions in different brain regions. In this study we aim to distinguish neural and vascular influences of a single dose of caffeine in measurements of task-related brain activity using simultaneous EEG–FMRI. We chose to compare low-level visual and motor (paced finger tapping) tasks with a cognitive (auditory oddball) task, with the expectation that caffeine would differentially affect brain responses in relation to these tasks. To avoid the influence of chronic caffeine intake, we examined the effect of 250 mg of oral caffeine on 14 non and infrequent caffeine consumers in a double-blind placebo-controlled cross-over study. Our results show that the task-related BOLD signal change in visual and primary motor cortex was significantly reduced by caffeine, while the amplitude and latency of visual evoked potentials over occipital cortex remained unaltered. However, during the auditory oddball task (target versus non-target stimuli) caffeine significantly increased the BOLD signal in frontal cortex. Correspondingly, there was also a significant effect of caffeine in reducing the target evoked response potential (P300) latency in the oddball task and this was associated with a positive potential over frontal cortex. Behavioural data showed that caffeine also improved performance in the oddball task with a significantly reduced number of missed responses. Our results are consistent with earlier studies demonstrating altered flow-metabolism coupling after caffeine administration in the context of our observation of a generalised caffeine-induced reduction in cerebral blood flow demonstrated by arterial spin labelling (19% reduction over grey matter). We were able to identify vascular effects and hence altered neurovascular coupling through the alteration of low-level task FMRI responses in the face of a preserved visual evoked potential. However, our data also suggest a cognitive effect of caffeine through its positive effect on the frontal BOLD signal consistent with the shortening of oddball EEG response latency. The combined use of EEG–FMRI is a promising methodology for investigating alterations in brain function in drug and disease studies where neurovascular coupling may be altered on a regional basis.

## Highlights

► Caffeine reduced task related BOLD responses in visual and motor cortex. ► However, amplitude and latency of visual evoked potentials remained unaltered. ► Performance and frontal BOLD response in an auditory oddball task were enhanced. ► Correspondingly, caffeine reduced the latency of target-evoked potentials. ► EEG–FMRI can separate vascular and neural influences of caffeine in low consumers.

## Introduction

In the brain caffeine acts as a nonselective antagonist of A_1_ and A_2A_ adenosine receptors (Dunwiddie and Masino, 2001). Increased neuronal activity is mediated through action on A_1_ and A_2A_ adenosine receptors, while vasoconstriction and as a consequence, reduction in cerebral blood flow (CBF), is mediated through action on A_2A_ receptors. Caffeine, therefore, can have both neural and vascular effects depending on the ratio of A_1_ and A_2A_ receptors in different brain regions (Chen and Parrish, 2009a; Laurienti et al., 2003). In addition, there is also evidence showing that caffeine has secondary effects on other neurotransmitter systems including dopamine, acetylcholine and noradrenaline (Ferre, 2008; Fredholm et al., 1999; Latini and Pedata, 2001).

Previous studies (Mulderink et al., 2002) have investigated the effect of caffeine on resting and sensorimotor BOLD activation and have demonstrated that caffeine decreases resting perfusion (Cameron et al., 1990) consistent with its vasocontrictive effect. Caffeine can alter the BOLD contrast arising from task-related changes in brain activity. The direction of modulation, however, varies among studies (Laurienti et al., 2003; Liau et al., 2008; Mulderink et al., 2002). The complex influence of caffeine is likely to arise from a combination of altered baseline blood flow, and hence modified baseline BOLD signal from which changes are measured, changes in the brain's resting metabolism and altered coupling between task-related changes in blood flow and metabolism (Chen and Parrish, 2009b; Griffeth et al., 2011). These factors are likely to be further influenced by the caffeine dose administered (Chen and Parrish, 2009a). It has been argued that the magnitude of the BOLD signal can decrease or increase due to caffeine, because of a combination of vascular and neural influences, the net effect of which depends on factors such as receptor number and affinity (Laurienti et al., 2003). Laurienti et al. (2002) demonstrated that the effect of caffeine on the changes in the BOLD signal depends on the individual habitual caffeine intake of the volunteers taking part in the study, with greater positive BOLD signal changes being observed in high compared to low caffeine users (Laurienti et al., 2002).

A few FMRI studies have investigated the effects of caffeine on cognitive functions (Bendlin et al., 2007; Koppelstaetter et al., 2008). For example, Koppelstaetter et al. (2008) showed that caffeine selectively modulated the BOLD signal in frontal cortex during a verbal working memory task in a sample of acutely caffeine abstinent individuals; there was a significant caffeine effect on the bilateral medial frontal cortex extending to the right anterior cingulate. These brain regions are associated with attentional and executive functions such as motivated attention, error detection, planning and problem solving (Carter et al., 1999; Lawrence et al., 2003; Liu et al., 2003).

Electrophysiological studies of the effect of caffeine on the central nervous system have demonstrated spectral shifts towards higher frequency. A significant reduction after caffeine administration of power in the lower alpha or theta bands (6–10 Hz) has been observed by several studies (Barry et al., 2005; Bruce et al., 1986; Dimpfel et al., 1993). This evidence supports the neural stimulating effects of caffeine but effects on specific cognitive activities cannot easily be distinguished using resting-state electroencephalography (EEG) measures. Event-related potentials (ERPs), however, are more appropriate to investigate the effects of caffeine on the organisation and timing of cognitive processes in the brain during performance of a specific task. The effects of caffeine on cognitive and motor function have been examined in several investigations (Deslandes et al., 2005; Rees et al., 1999; Ruijter et al., 2000a) in an attempt to clarify possible mechanisms involved. Caffeine's effects on attention, mood and alertness have been reported (Rogers and Smith, 2011; Rogers et al., 2010; Ruijter et al., 2000a, 2000b). In addition, changes in the latency and amplitude of the ERP component, P300, (Deslandes et al., 2004; Pan et al., 2000; Ruijter et al., 2000a) have been related to the modulatory effects of caffeine.

The present study investigated the neurocognitive effects of a single dose of caffeine using simultaneous EEG–FMRI in an attempt to identify both general vascular and specific neural effects of caffeine. The study aimed to determine the effect of caffeine on non and infrequent caffeine consumers during performance of visual, motor and cognitive (auditory oddball) tasks. Non and infrequent consumers were selected to avoid influences of the effects of frequent caffeine intake (Rogers et al., 2010). As has been shown by Lorist et al. (1994) the cognitively beneficial effect of caffeine is larger in a task condition in which the targets are temporally unpredictable, hence the choice of auditory oddball (Lorist et al., 1994). We used visual and (paced) motor tasks as control tasks to identify general vascular influences and thus identify the specificity of effects of caffeine on brain responses in relation to cognitive performance. In order to monitor the neurophysiological responses we employed simultaneous FMRI and EEG enabling us to perform measurements of cerebral haemodynamic response and cortical electrical activity. Whilst the neurocognitive effects of caffeine have been investigated using these techniques independently, they have not previously been utilised in combination to assess such effects in the same subject and in the same environment. In addition to EEG and BOLD FMRI, arterial spin labelling measurements of cerebral blood flow were obtained to indicate caffeine's influence on the perfusional state of the brain.

Based on recent evidence (Koppelstaetter et al., 2008) we hypothesized that caffeine might selectively, in comparison with the visual sensory and the motor task, increase the task-related BOLD signal in frontal cortex associated with the effects of caffeine on mental performance related to attention and executive functioning during the auditory oddball task. Alongside this we anticipated that caffeine may have a differential effect on the event related potentials (ERPs) elicited by the oddball task, compared to the low-level sensory (visual) task.

## Materials and methods

### Participants

Fourteen healthy, non or infrequent (≤ 36 mg/day, equivalent to the caffeine present in a single cup of tea) caffeine consumers, right handed male volunteers (aged 20–32 years, mean age 25.7 ± 4) were recruited for the study under the following inclusion criteria: no history of diabetes, brain injuries, hypertension, any psychiatric or neurological disease, alcohol or drug abuse, no use of tobacco products. Information on participants' caffeine intake over the 8 weeks preceding testing was recorded during the week preceding testing using a caffeine intake questionnaire that assessed the frequency of consumption of chocolate, teas, coffees, colas and other caffeine-containing drinks and dietary supplements (Heatherley et al., 2006b). Weekly caffeine intake was calculated from these self-report data using dietary and manufacturers' information on caffeine content (Heatherley et al., 2006a), and this was confirmed with participants on arrival at each FMRI session. The study was approved by the local ethics committee. Written informed consent was obtained from each participant. All participants were free of medication at the time of scan sessions.

### Experimental design and caffeine administration

Caffeine is absorbed in the stomach and small intestine. The peak concentration in plasma is reached 30–90 min after oral ingestion and the half-life of caffeine varies from 4 to 8 h depending on body mass, age, concurrent medications and liver function. The hydrophobic properties of caffeine allow its passage through all biological membranes. The blood brain barrier is permeable to caffeine and, after reaching peak absorption, brain levels of caffeine remain stable for approximately 60–80 min (Fredholm et al., 1999; Nehlig and Boyet, 2000).

**Fig. 1:**
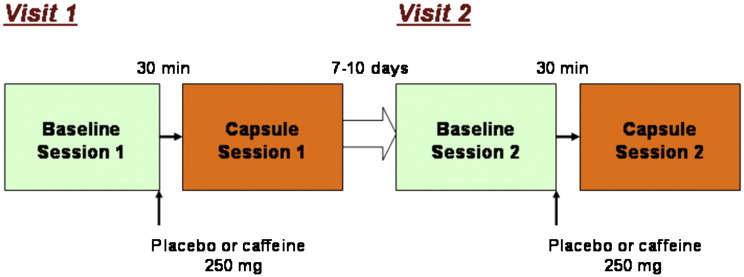
Experimental design. Each participant was scanned twice (baseline followed by caffeine/placebo scan) on each of two separate visits at the same time of the day at least one week apart. On each visit participants were scanned once with the stimulus paradigm described below (BP baseline placebo or BC baseline caffeine), removed from the magnet at which point they received an oral dose of either a gelatine capsule containing 250 mg caffeine or placebo (cornflour) then scanned again 30 min later (scans DC or DP respectively). Caffeine was given in a double-blind, crossover placebo-controlled, manner.

Each participant was scanned twice (baseline placebo/caffeine, BP/BC respectively, followed by a caffeine/placebo scan, DC/DP respectively) on each of two separate visits at the same time of the day at least one week apart. On each visit participants were scanned once with the stimulus paradigm described below, removed from the magnet at which point they received an oral dose of either a gelatine capsule containing 250 mg caffeine or placebo (cornflour) then scanned again 30 min later (see Fig. 1). Caffeine was given in a double-blind, crossover, placebo-controlled, manner. A moderate caffeine dosage (250 mg, equivalent to the caffeine present in 2 cups of ground coffee) was chosen to reflect caffeine intake of common drinking habits. At the end of the second scanning session participants were asked to guess on which day they received caffeine and on which day they received placebo.

### Physiological parameters

Systolic and diastolic blood pressure (mm Hg, non-invasive blood pressure cuff) and heart rate were recorded at the beginning and at the end of every scanning session. Partial pressure of end-tidal carbon dioxide (Pet_CO_2__, mmHg) was measured during the MRI session. Each volunteer wore a nasal cannula through which respiratory gases were sampled (capnograph models CD-3A, AEI Technologies, Pittsburgh, PA, USA). Continuous logging of the P_CO_2__ waveform (CED 1401, Spike 2 Software, CED, Cambridge UK) was performed and Pet_CO_2__ was calculated for each breath using Matlab (The MathWorks Inc. Natick, MA). Mean Pet_CO2_ was calculated for each subject during an 8-minute period without stimulus presentation towards the beginning of each scan session.

### Tasks

Participants were presented with 3 different tasks: a visual task, a simple paced-motor (finger tapping) task and an auditory oddball task while simultaneous EEG and whole-brain FMRI recordings were made. Participants were trained on the tasks before entering the scanner.

#### Visual task

A black and white square checkerboard, reversing at 4 Hz, with a red central fixation cross was presented in a block design with five repeats of 40 s stimulation and 20 s of rest. The rest condition was a dark screen with the same fixation cross. Duration of the task was 5 min and 15 s.

#### Finger tapping task

A visually cued finger tapping task was employed for motor activation. The paradigm was presented in a block design of five repeats of 26 s tapping and 26 s rest, duration was 4 min and 45 s. Participants were instructed to tap the fingers of their right hand on buttons on the MR compatible response device cued by a series of numbers (1 to 5, representing the first digit (thumb) to the fifth digit) which appeared on the display screen after the command “Tap”. The rate of the paced tapping was 1 tap per second. During the rest condition, followed by command “Rest,” the same numbers (1 to 5) were presented on the screen, but participants were asked just to look at the presented numbers without tapping.

#### Auditory ‘oddball’ task

A three-stimulus continuous (20 min) auditory ‘oddball’ task was utilised to elicit a cognitive response. The frequent standard auditory stimuli (1 kHz, lasting 100 ms), the rare target auditory stimuli (1.5 kHz, lasting 100 ms) and the novel auditory stimuli consisting of noises (e.g. dog barks, whistle, etc.), were presented randomly via headphones (NordicNeuroLabs electrostatic headphones) with a mean interstimulus interval (ISI) of 2.05 s (randomized in the range 1.8 to 2.3 s). A total of 576 stimuli were delivered. The target and novel stimuli each occurred with a probability of 15% of trials, whilst the standard stimuli occurred with a probability of 70%. Participants were asked to respond quickly and accurately to the target stimuli only by pressing the right index finger button on the MR compatible response device and to ignore standard or novel stimuli. Behavioural measures were assessed during the performance of the auditory oddball task as the time taken to respond to target stimuli (reaction time for correct responses in ms), number of false alarms and missed responses (misses).

All tasks were programmed using the integrated software tool “Presentation” Version 11.3 (http://www.neurobs.com). Stimulus presentation was triggered by the MR-scanner.

### Perfusion measurement

MRI was conducted on a General Electric Excite HDx 3T MRI scanner using an eight-channel receive-only head coil. Preceding the functional tasks, whole brain cerebral blood flow (CBF) was measured using pulsed arterial spin labelling (PASL) with a proximal inversion and control for off-resonance effects (PICORE), quantitative imaging of perfusion using a single subtraction (QUIPSSII), sequence with gradient-echo echo-planar imaging (GE-EPI) readout (Wong et al., 1998). Seventy-one tag-control image pairs with 16 axial slices (3.75 × 3.75 × 7 mm voxel resolution, matrix 64 × 64, with a 1 mm inter-slice gap) were acquired (TR/TE = 2200/19.8 ms, single inversion time with TI1 = 700 ms at TI2 = 1350 to 2150 ms from the most proximal to the most distal slice respectively. A separate single shot EPI (M_0_) scan was acquired (TR = ∞) with the same parameters to measure the equilibrium brain tissue magnetisation for calibration purposes. The signal from deep white matter was measured and the M_0_ of blood was estimated from the white matter signal assuming a ratio of proton density of blood to that in white matter of 1.06 (Wong et al., 1998) and T1 blood = 1.7 s (Lu et al., 2004), T2* blood = 0.1 s (Silvennoinen et al., 2003), T2* white-matter = 0.047 s (Wansapura et al., 1999). In-house software was used to convert the estimate of M_0_ of blood and the mean difference of the motion-corrected (MCFLIRT (Jenkinson et al., 2002)) tag-control perfusion pair time-series to CBF in ml/100 g/min by applying a standard single compartment model (Wong et al., 1998). Following registration to the common standard space of the Montreal Neurological Institute (MNI), region of interest CBF values were extracted for each individual for global grey matter (defined from the probabilistic map of tissue priors available in the FMRIB software library, www.fmrib.ox.ac.uk/fsl) and regions identified from the significant caffeine effect on task-related BOLD signal responses from the visual, motor and auditory tasks.

### BOLD FMRI data acquisition

FMRI was performed using GE-EPI with repetition time (TR) = 3000 ms, echo time (TE) = 35 ms, flip angle (α = 90°), field of view = 205 mm, 50 slices parallel to inter-commissural (AC-PC) plane, matrix size = 64 × 64, slice thickness = 3.2 mm, the slices were contiguous (zero slice gap) with a voxel size of 3.2 × 3.2 × 3.2 mm^3^ covering the whole brain. A 1 × 1 × 1 mm^3^ *T1* weighted structural scan was acquired to facilitate registration of the functional data to the common standard space of the MNI.

### EEG data acquisition

Continuous EEG data were collected from 30 standard scalp electrodes using the BrainAmp MR, a high-input impedance amplifier specifically designed for recordings in high magnetic fields (BrainProducts, Munich, Germany). Sintered Ag/AgCl ring electrodes with built-in 5 kΩ resistors mounted into an electrode cap according to the 10–20 system (Falk Minow Services, Herrsching, Germany) were used. One additional electrode was placed below the left eye and one on the lower back to monitor eyeblinks and electrocardiogram, respectively. Electrode impedances were maintained below 10 kΩ before recording began. The MR compatible EEG amplifier was fixed beside the head coil and powered by a rechargeable power pack. The subject's head was immobilized using sponge pads. The amplified EEG signals were transmitted via a fibre optic cable to a recording personal computer placed outside the scanner room. All 32 channels were recorded with FCz as reference. The data were recorded with a passband of 0.016–250 Hz and a sampling rate of 5 kHz.

### FMRI data analysis

BOLD FMRI data were analysed for each subject using the FMRIB Software Library (FSL) version 5.98 (www.fmrib.ox.ac.uk/fsl). Prior to statistical analysis of BOLD data the following processing was applied to each subject's time series of FMRI volumes: brain extraction using BET, motion correction using MCFLIRT (Jenkinson et al., 2002), spatial smoothing using a Gaussian kernel of full width at half maximum 5 mm, and nonlinear high-pass temporal filtering (Gaussian weighted least squares straight line fitting, with high-pass filter cut-off of 90 s for visual and finger tapping tasks and 60 s for auditory oddball). Single-subject low-resolution functional images were co-registered to their corresponding high-resolution structural images, and then co-registered to a standard brain (Montreal Neurological Institute — 152 template) using FLIRT (Jenkinson and Smith, 2001).

Single-subject time-series statistical analysis was carried out using a general linear model (GLM) approach with local autocorrelation correction. The design matrix was generated using a single gamma haemodynamic response function (HRF) and its first (temporal) derivative. For visual and finger tapping tasks the block functions were convolved with canonical HRF to generate the model time course for the conditions (box-car design). For the auditory ‘oddball’ task the design matrix consisted of an onset vector of target stimuli, novel stimuli and nontarget stimuli (event related design) also convolved with the canonical HRF. In addition six rigid-body motion correction parameters were entered as confounding covariates to mitigate the influence of head motion in the data.

Data from each participant were combined in second (group) level mixed-effects models using FLAME with automated outlier de-weighting (Beckmann et al., 2003; Woolrich et al., 2004). To identify the distribution of activity in each task without influence of caffeine, scans from baseline placebo (BP, pre-dose) and baseline caffeine (BC, pre-dose) scans were combined at the second level to produce group average baseline activity maps. The effects of caffeine were identified from a paired second level analysis separately for each task: visual, motor and auditory oddball. These analyses modelled the interaction of the effect of “dosing”, namely baseline pre-dose (B) or post-dose (D), and the effect of “drug”, namely placebo (P) or caffeine (C). The interaction is described by the contrast (DC–BC)–(DP–BP) and represents caffeine's effects controlled by baseline scans. Both positive and negative interaction effects were examined. A cluster correction for multiple comparisons was used, with an initial threshold of Z > 2.3, and a whole-brain corrected cluster threshold of p < 0.05 for each condition (Worsley, 2001).

Within the regions of significant caffeine-related changes in task-related BOLD response, percentage task-related BOLD signal changes were calculated (featquery, www.fmrib.ox.ac.uk/fsl) in each condition BP, BC, DP and DC and time-course BOLD signal responses were extracted and plotted for the visual and motor tasks. The time (T_50_) to reach 50% of the maximum response for the visual and motor tasks was estimated for each subject (Liu et al., 2004) following linear interpolation of the region of interest BOLD time-series data to a new sampling interval of 0.1 s and averaging across stimulus cycles. The T_50_ was tested for an effect of caffeine using 2-way repeated measures analysis of variance. Identification of the T_50_ was not feasible for the auditory task because of the smaller BOLD signal changes in response to the presentation of brief events and hence lower signal to noise ratio of the BOLD responses.

### EEG data analysis

Brain Vision Analyzer software (Brainproducts, Germany) was used for correction of MR gradient and ballistocardiographic (BCG) artefacts (Allen et al., 1998, 2000). Gradient artefacts were removed as implemented in Vision Analyzer software by subtracting an artefact template from the data, using a baseline-corrected sliding average of 20 consecutive volumes. Following scanner artefact removal, pulse artefact subtraction was applied. This procedure works analogously by averaging EEG signal synchronized to the ECG. Segments contaminated by artefacts due to gross movements were removed following visual inspection (maximum total 20% rejected from the data in a given subject). After removal of any bad channels, all channels were re-referenced to common average. In order to eliminate slow drifts and high frequency noise, EEG data were then filtered with a 0.2 Hz high-pass filter and a 30 Hz low-pass filter. The filters used were phase-shift-free Butterworth filters with a 24 dB/octave slope. ICA was performed on the continuous EEG data using the infomax algorithm and components representing eye-blink were removed from the data (Srivastava et al., 2005). The data were then segmented into stimulus-locked segments (for the visual stimulation 100 ms before to 300 ms after stimuli occurred and for the auditory oddball task 200 ms before to 900 ms after target, novel or non-target stimuli occurred). Automatic artefact rejection was then performed before response averaging to reject trials contaminated by residual artefacts. Specifically, trials with a difference of 100 μV or greater between the largest negativity and largest positivity, and trials with 60 or more data points in a row with the same value were rejected automatically. All segments were then baseline corrected using prestimulus data. Finally, segments were averaged within trial types to produce single-participant averages, and single-participant averages were then averaged to produce grand-averages.

### Statistical analyses

Values are quoted as mean ± standard deviation. Physiological, behavioural, regional CBF and summary event related potential (ERP) parameters were analysed using a 2-way repeated-measures (within subject) analysis of variance (ANOVA) to isolate the caffeine effect while controlling for between day variations with the inclusion of the baseline scans. The main factors in the ANOVA were dosing within scan session i.e. baseline (B) or post-dose (D) scan and drug i.e. placebo (P) or caffeine (C). The drug effect of interest is given by the interaction of factors “dosing” × “drug.” Post-hoc two-tailed paired t-tests were also applied after ANOVA.

## Results

### Physiological parameters

The bodyweight of recruited volunteers was 76.5 ± 11 kg (mean ± standard deviation) with a range of 57 kg to 100 kg. This gives a mean dose of 0.306 ± 0.0450 mg/kg with a range from 0.228 mg/kg to 0.4 mg/kg.

Caffeine significantly increased systolic blood pressure (2-way repeated-measures ANOVA) (Table 1). Although the interaction effect in the 2-way ANOVA did not reach significance for diastolic blood pressure and heart rate, post-hoc comparisons of DC with BC and DP suggest an increase in both parameters with caffeine administration. There was no significant correlation between bodyweight and changes in blood pressure and heart rate between the baseline caffeine (BC) and caffeine (DC) conditions. Good quality Pet_CO2_ recordings were obtained for both scans and both sessions in 9 subjects. There was a significant effect of caffeine in reducing Pet_CO2_ (Table 1).

Eight participants correctly guessed the order in which they received caffeine and placebo, while six participants did not (not significantly different from chance).

### Behavioural performance for the ‘oddball’ task

The 2-way repeated-measures ANOVA (within-subjects) analysis revealed that caffeine significantly reduced the number of missed responses to the target oddball stimulus (Table 2). However there was no significant effect of caffeine on the number of false alarms or on reaction times (Table 2).

### FMRI: visual task

**Fig. 2:**
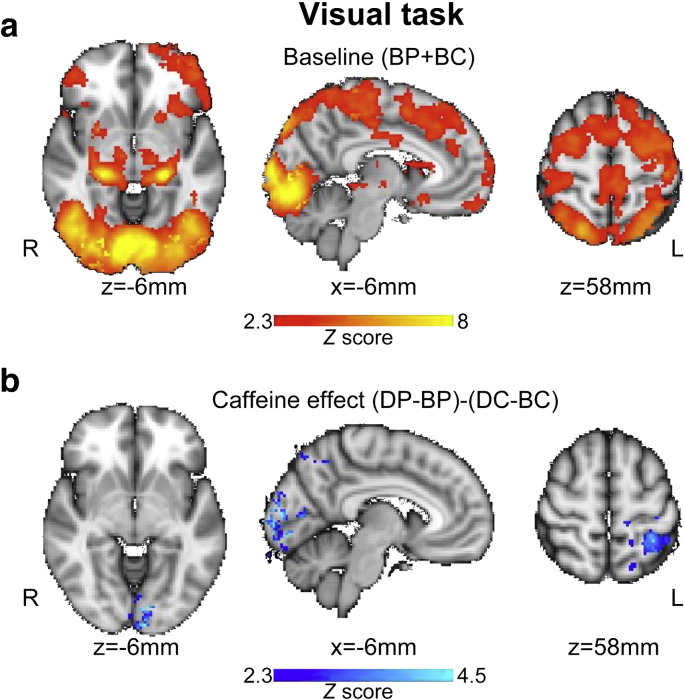
Significant BOLD signal changes in response to the visual task vs, rest. a) shows the group mean visual task related activity (positive signal change) for the two baseline scans combined (BP and BC). b) shows the baseline-controlled group difference between placebo and caffeine administration ((DP–BP)–(DC–BC)) indicating a significant reduction in the visual BOLD response. Signal changes were deemed significant at *Z* > 2.3 with a whole brain cluster based correction at p < 0.05.

The visual stimulation elicited widespread activation (a BOLD response) in visual cortex including the occiptal pole, occipital fusiform gyrus, intracalcarine cortex and lateral occipital cortex (Fig. 2a shows baseline conditions BP and BC combined). Beyond visual cortex, the task induced BOLD signal increases vs. rest in bilateral hippocampus, thalamus, frontal pole, paracingulate gyrus, premotor cortex, pre- and post-central gyrus and superior parietal lobule. The second level within-subjects analysis examining the caffeine effect while controlling for baseline differences ((DP–BP)–(DC–BC)) revealed that the BOLD signal change in visual cortex was significantly reduced by caffeine (Fig. 2b). The peak statistical difference was seen at location (MNI co-ordinates) x = − 6, y = − 98, z = − 6 mm, in primary visual cortex. A second cluster of voxels of reduced BOLD signal change lay in the left superior parietal lobule (Fig. 2b).

### FMRI: finger tapping task

**Fig. 3:**
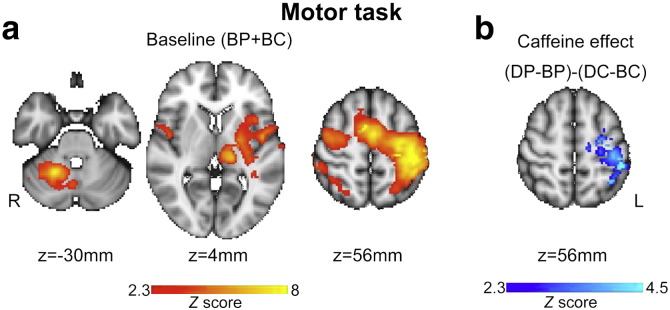
Significant BOLD signal changes in response to the motor (finger tapping) task vs rest. a) shows the group mean motor task related activity (positive signal change) for the two baseline scans combined (BP and BC). b) shows the baseline-controlled group difference between placebo and caffeine administration ((DP–BP)–(DC–BC)) indicating a significant reduction in the BOLD response. Signal changes were deemed significant at *Z* > 2.3 with a whole brain cluster based correction at p < 0.05.

The finger tapping task elicited activation in the left sensorimotor cortex, supplementary motor area, thalamus, putamen and right superior cerebellum (Fig. 3a shows baseline conditions BP and BC combined). The second level within-subjects analysis examining the caffeine effect ((DP–BP)–(DC–BC)) revealed that the BOLD signal change in left sensorimotor cortex was significantly reduced by caffeine (Fig. 2b). The peak statistical difference was seen at location (MNI co-ordinates) x = − 50, y = − 38, z = 56 mm.

The mean task-induced BOLD percentage signal changes for each scan are shown in Fig. S1a and b for the regions in which caffeine demonstrated a significant effect for the visual and motor tasks respectively. The time-course responses for the block-design tasks, visual and motor, are illustrated in Figs. S2 and S3. There was no significant caffeine effect on the time (T_50_, not shown) for the BOLD response to visual and motor stimulation in these regions to reach 50% of its maximum (p > 0.5 for the interaction effect in 2-way repeated measures ANOVA).

### FMRI: auditory oddball task

**Fig. 4:**
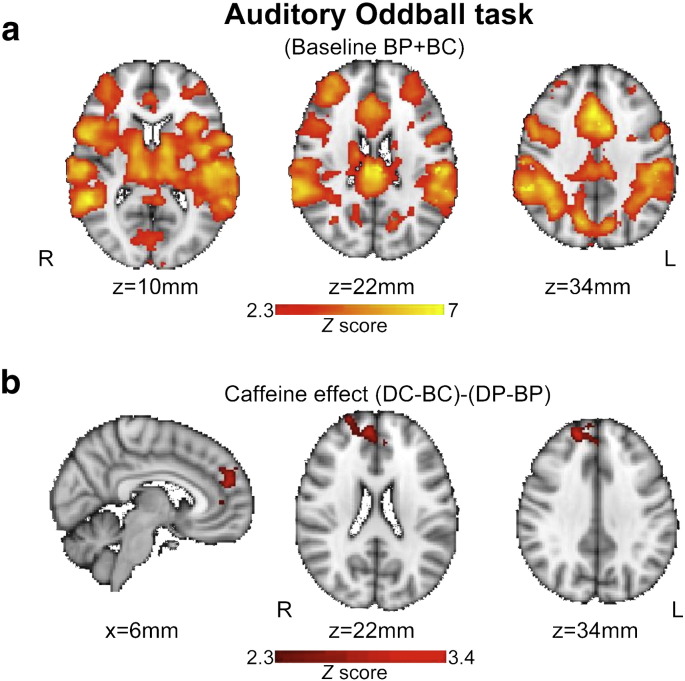
Significant BOLD signal changes during the auditory oddball task (target stimulus vs non-target stimulus). a) shows the group mean motor task related activity (positive signal change) for the two baseline scans combined (BP and BC). b) shows the baseline-controlled group difference between caffeine and placebo administration ((DC–BC)–(DP–BP)) indicating an increase in BOLD signal with caffeine. Signal changes were deemed significant at *Z* > 2.3 with a whole brain cluster based correction at p < 0.05.

Comparison of the target stimuli versus the non-target stimuli was associated with significant hemodynamic activity (shown in Fig. 4a for BP and BC scans combined) consistent with previous studies (Kiehl et al., 2001, 2005; Polich, 2003). Supplementary Tables 1S, 2S and 3S, list the regions in which comparisons target–non-target, target– novel and novel–non-target yielded significant responses in the baseline scans at the group level. The experimental condition target–non-target was of primary interest in our analysis, representing the response to oddball stimuli.

The effect of caffeine in the group level comparison controlling for baseline variation ((DC–BC)–(DP–BP)) was manifested in the target vs. non-target experimental condition. There was a significantly more positive BOLD response for target vs. non-target stimuli induced by caffeine in superior frontal gyrus, frontal pole and paracingulate gyrus (Fig. 4b). The peak statistical difference was seen at location (MNI co-ordinates) x = 18, y = 50, z = 34 mm. There was no significant effect of caffeine, either (DC–BC)–(DP–BP) or (DP–BP)–(DC–BC), on the experimental conditions target–novel and novel–non-target.

There was no significant correlation between bodyweight and the difference in percentage BOLD signal change between baseline caffeine (BC) and caffeine (DC) conditions, within the regions in which the significant effect of caffeine was observed for each of the three tasks, visual, motor (finger tapping) and auditory “oddball”.

The mean task-induced BOLD percentage signal changes for each scan are shown in Fig. S1c for the regions in which caffeine demonstrated a significant effect for auditory oddball (target vs. non-target) task.

### EEG: visual task

**Fig. 5:**
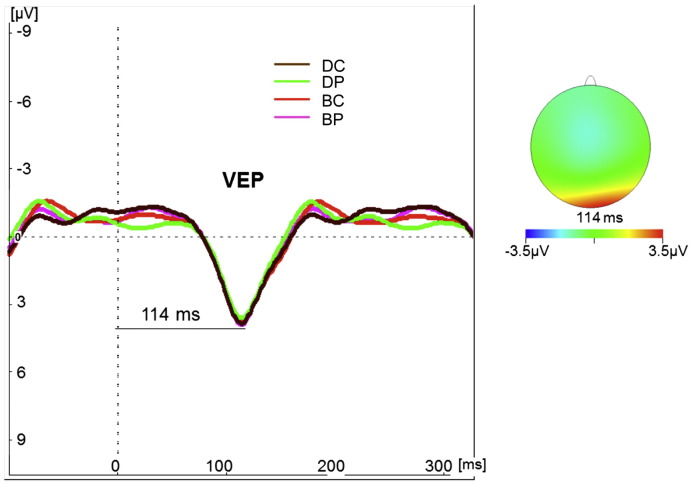
Group mean visual evoked potentials (VEPs) recorded during baseline placebo (BP), baseline caffeine (BC), placebo (DP) and caffeine (DC) conditions (left). The dotted vertical line marks stimulus presentation time. The scalp map for the grand-average VEP (right). The time point shown is at the peak of the VEP for the participants performing the visual task (latency 114 ms). The magnitude is indicated by the colour-bar scale at the bottom.

The occipital electrodes O1 O2 and Oz were used for visual evoked potential (VEP) analysis. The amplitude was defined as peak-to-peak difference between the global minimum in the interval 25–90 ms and the global maximum in the interval 80–140 ms. The latency was defined as the time from stimulus presentation to the point of maximum positive amplitude within the time window (Fig. 5). Two-way repeated measures ANOVA to compare the amplitudes and latencies of evoked potentials revealed no significant effect of caffeine through the interaction “dosing” × “drug” (p > 0.05) (Table 3).

### EEG: auditory “oddball” task

**Fig. 6:**
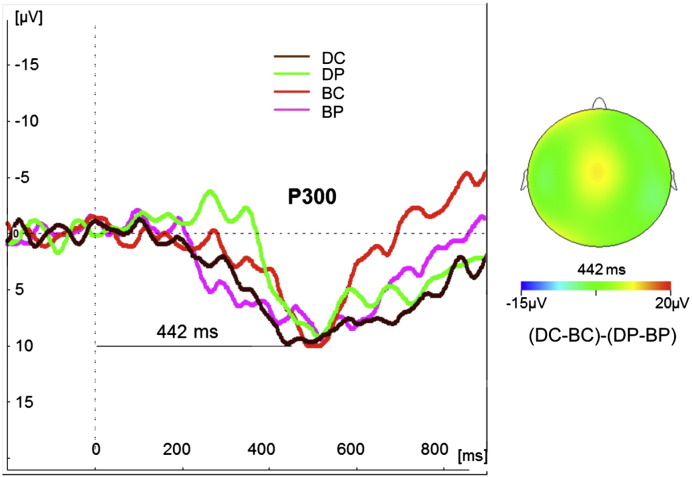
Group mean evoked response waveforms for the auditory target stimulus, P300, recorded during baseline placebo (BP), baseline caffeine (BC), placebo (DP) and caffeine (DC) conditions (left). The dotted vertical line marks stimulus presentation time. The group mean scalp map is shown (right) for the baseline-controlled group difference between caffeine and placebo administration ((DC–BC)–(DP–BP)) at the peak latency of the response on caffeine (DC) (442 ms).

The midline electrodes Fz, Cz and Pz were used for analysis (Tables 4 and 4S). The response for the target stimulus, the P300 amplitude, was defined as the largest positive peak (relative to the 100 ms pre stimulus baseline) within the time window of 300–550 ms post stimulus presentation (Fig. 6). The response for the novel stimulus, “novelty P300” amplitude was defined as the largest positive peak (relative to the 100 ms pre stimulus baseline) within the time window of 250–500 ms post stimulus presentation. The response for nontarget stimuli, P200, amplitude was defined as peak-to-peak difference between the minimum in the interval 90–200 ms and the maximum in the interval 200–450 ms. A scalp map of the caffeine effect on the “novelty P300” response (comparison (DC–BC)–(DP–BP)) is shown in Fig. 6, indicating a fronto-central positivity at a latency of 442 ms.

The two-way repeated measures ANOVAs applied to event related potentials revealed a significant effect of caffeine on the ERP latency evoked by target stimuli (p < 0.05 for the interaction effect for all three midline electrodes (Table 4)). However, there was no significant effect on the amplitude at these electrodes. Furthermore, there was also no effect of caffeine on amplitude and latency of evoked potentials elicited by non-target or novel stimuli (Table 4S).

There was no significant correlation between bodyweight and the differences in Target stimulus ERP latency (electrode Cz tested) between baseline caffeine (BC) and caffeine (DC) conditions.

For each subject a number of stimulus trials was excluded for each subject from the EEG data because of the signal cleaning procedure described above. There was no significant caffeine effect on the number of visual evoked or auditory oddball trials incorporated in the EEG analysis (p > 0.05 for a 2-way repeated measures ANOVA on trials remaining after EEG cleaning).

### Reductions in perfusion

Arterial spin labelling measurements indicated that cerebral blood flow was significantly reduced across grey matter by caffeine administration (2-way repeated-measures ANOVA, interaction effect p < 0.001). Comparison of the baseline caffeine (BC) with the caffeine condition (DC) showed a mean reduction of 19% in grey matter. Perfusion changes were further tested in regions of interest defined functionally by the significant caffeine effects on the BOLD responses in the visual, the motor (finger tapping) and the auditory “oddball” task described above. These regions respectively showed 30% (visual cortex, left superior parietal lobule), 34% (left sensorimotor cortex) and 24% (frontal pole, superior frontal gyrus and paracingulate gyrus) reductions in CBF (BC vs DC conditions). CBF measurements are summarised in Supplementary Table 5S. There was no significant correlation (p < 0.05) between percentage reductions in global grey matter CBF (BC vs. DC conditions) and bodyweight of volunteers suggesting that the range of bodyweights did not lead to significant variation in drug effect, as indexed by CBF.

## Discussion

The present study investigated the effect of a single dose of oral caffeine on cognitive, visual and motor responses in non and infrequent caffeine consumers. Adopting a multimodal approach of simultaneous EEG–FMRI enabled the acquisition of functional datasets of both good spatial and temporal resolution and under the assumption of a coupling between observable EEG and FMRI responses, allowed us to demonstrate likely changes, resulting from caffeine ingestion, in the relationship between task-related electrophysiological activity and the cerebral haemodynamic response.

In the brain, caffeine is a non-selective antagonist of adenosine receptors. Its blocking action of A_1_ and A_2A_ adenosine receptors has a neuronal excitatory effect (Dunwiddie and Masino, 2001) since normally adenosine acts to inhibit release of excitatory neurotransmitters (Fredholm et al., 1999). Caffeine has a second effect of blocking A_2A_ adenosine receptors in the cerebral blood vessels, resulting in vasoconstriction (Dunwiddie and Masino, 2001). Conventional BOLD FMRI signals are dependent on a change in oxygen consumption and blood flow. Caffeine has the potential therefore to influence the BOLD signal in a complex manner through alterations in neuronal activity and the vascular responsiveness to such alterations.

### FMRI findings

The vasoconstrictive influence of caffeine in our low-consuming cohort is evident through the observed widespread reductions in cerebral blood flow (19% across grey matter). This reduced perfusion is consistent with previous observations using arterial spin labelling techniques (Field et al., 2003; Laurienti et al., 2003). In the motor and visual cortices with caffeine administration we observed reduced task-induced BOLD responses. However, in frontal areas we observed a more positive task-induced BOLD signal arising from the auditory oddball task. This would be consistent with an enhancing effect of caffeine on brain areas associated with executive functions. We consider these regional alterations in BOLD response in the context of previous studies of altered neurovascular coupling with caffeine.

The overall effect of caffeine on the BOLD signal is likely to depend on which of the two receptor system effects (A_1_ vs. A_2_) dominates. In subjects or brain regions in which the excitatory effect on task-related neuronal activity dominates we might expect an enhanced task-induced BOLD response. Where the vasoconstrictive effect dominates we might expect a diminished BOLD response. This variability of caffeine's influence on the receptor systems has been suggested as an explanation for differential effects of caffeine on the BOLD response according to daily dietary caffeine intake (Laurienti et al., 2002). Laurienti et al, 2002 suggest that low caffeine users experience more vasoconstriction than neural excitation, with A_2_ effects dominating, resulting in an observed reduced visual BOLD response in low caffeine users that is consistent with our present observations from the visual and motor task. Laurienti et al, 2002 further suggest that neural A_1_ receptors tend to increase more than A_2_ receptors with high caffeine use resulting in enhanced neural responses and therefore dominating the vasocontrictive influence. This effect may explain the enhanced visual BOLD response observed in high caffeine users (Laurienti et al., 2002) and is consistent with other studies in which caffeine-consuming volunteers show increases in BOLD signal contrast (Mulderink et al., 2002). Differences between studies in the caffeine modulation of BOLD responses may also be compounded by differences of dose, as non-linear dose effects on BOLD amplitude have been demonstrated (Chen and Parrish, 2009a). Extending these explanations of caffeine response variability, we suggest that relative regional differences of caffeine's effect on the task-induced neural and vascular responses, mediated by the different receptor sub-systems, may explain the enhancement of the BOLD response in the frontal cortex and diminution in the motor and visual cortices in the present study. Indeed, regional differences in caffeine's neural and vascular action are likely as levels of adenosine A_2A_ receptors, responsible for the vasoconstrictive effect of caffeine appear, relative to widely distributed adenosine A_1_ receptors, to be lower in the prefrontal or frontal cortex (Bauer et al., 2003; Fastbom et al., 1987; Svenningsson et al., 1997).

The effect of caffeine on the regional BOLD response to stimulation can also be considered from the point of view of underlying cerebral blood flow and metabolism which give rise to BOLD image contrast. Recent studies have examined flow-metabolism coupling in a range of cerebral physiological states in which blood flow is modified (Ances et al., 2009; Brown et al., 2003) and specifically investigated the influence of caffeine (Chen and Parrish, 2009b; Griffeth et al., 2011). The investigations concerned visual and motor cortex in cohorts containing regular caffeine consumers who were asked to abstain from caffeine before the study. Both studies show a decrease in *n*, the coupling ratio between stimulus-induced fractional changes in CBF and CMRO_2_, and thus the importance of the underlying physiological responses. Griffeth et al, 2011, showed that consideration of absolute changes is important. They showed in visual cortex a caffeine induced reduction in baseline CBF, a reduction in task-induced increase in CBF, an increase in baseline CMRO_2_ and an increase in task-induced increase in CMRO_2_. These observations are consistent with the neural (CMRO_2_) and vasocontrictive (CBF) caffeine effects discussed in association with A_1_ and A_2A_ receptors. The net effect observed by Griffeth et al was no overall caffeine-induced change in visual stimulus-induced BOLD signal. However, the study makes clear that the modulation of the task-related BOLD response is highly sensitive to the balance of caffeine-induced changes in underlying and task-related changes in physiology. The BOLD response could be increased or decreased depending on the relative modulation of the CMRO_2_ and CBF response to stimulation. A slightly more attenuated CBF response would have resulted in a reduction of the overall BOLD response, as we see in the present study for our visual and motor tasks. Conversely a slightly less attenuated CBF response would have resulted in an increase of the overall BOLD response to stimulation, as we see for our oddball task. Given that in our present study we examine a low-consuming cohort and are comparing different brain regions it is likely that the balance of metabolic and perfusion changes may differ compared to earlier studies. We suggest that dose, cohort and importantly brain-region based differences in the absolute CMRO_2_ and CBF responses could contribute to the differential modulation of our observed BOLD responses between visual and motor cortices and frontal cortex.

### EEG findings

We examined two commonly reported features of the task-evoked potentials from the visual and auditory-oddball tasks, amplitude and latency. Scalp EEG does not reveal all features of evoked neuronal activity. We therefore interpret with caution the relationship between observed evoked potentials and the BOLD signal considering an empirically defined coupling between the two types of measurement of brain activity.

We saw no significant effect of caffeine on VEP latency or amplitude in any of the occipital electrodes. This is consistent with the study of Owen et al, 2001 (Owen et al., 2001) in an EEG study with a similar visual stimulus. However in the study by Azcona et al. (1995) it was shown that caffeine significantly increases amplitude of VEPs but has no effect on the latency. Such differences from the current experiment could be because of the visual task differences or the higher caffeine dose used by Azcona and colleagues. The unchanged VEPs combined with a reduced visual-evoked BOLD response suggest an altered empirical neurovascular coupling. This would be consistent with a preserved neural response and hence oxygen consumption but a reduced CBF response, consistent with the vasoconstrictive effect of caffeine on the task-induced absolute CBF response.

In the cognitive auditory oddball task we observed a shorter latency of the P300 evoked potential induced by target stimuli after caffeine administration and an improvement in task performance (significant reduction of missed responses). This altered latency is suggestive of caffeine induced alterations in neuronal activity. However, no change in evoked amplitude was observed. Our observed change in latency is consistent with previous examinations of caffeine's effects on neuronal activity. Discrimination between target and standard stimuli in an oddball task is believed to initiate frontal lobe activity that is sensitive to the attentional demands induced by task performance (Polich, 2007). P300 latency is negatively correlated with mental function in healthy subjects and shorter latencies indicate a superior cognitive performance (Polich, 2007; Polich and Herbst, 2000). Some studies have pointed to an improvement of cognitive performance after caffeine ingestion through a significant shortening of P300 latency, especially in the frontal cortex (medial frontal electrode Fz) (Deslandes et al., 2005; Kenemans and Lorist, 1995; Montenegro et al., 2005; Pan et al., 2000), consistent with the frontal distribution shown in the scalp maps of Fig. 6. Other studies have shown an increase in P300 amplitude, which we failed to find, with caffeine (Dixit et al., 2006; Kawamura et al., 1996; Ruijter et al., 2000a). However, observations of altered amplitude with caffeine are less consistent as some studies found no changes (Deslandes et al., 2005; Kenemans and Lorist, 1995) or even a decrease (Montenegro et al., 2005; Pan et al., 2000). Barry et al. (2007) suggest that in general, caffeine's effects on evoked responses are fairly focal and not widely distributed to all responses, the induced differences being more commonly found in later responses related to task performance and response production rather than global increases in ERP amplitude which might be expected from an increase in general arousal. This is consistent with the restriction of our observed caffeine effects to the P300 latency only for the target stimulus and with no detected effects on the other auditory stimuli or the visual evoked potentials.

The altered ERP and BOLD responses in the auditory oddball experiment occur in broadly similar (frontal) brain regions suggesting a possible link between the shortened latency and the enhanced frontal BOLD response. However, this must be interpreted with caution because BOLD and ERPs do not necessarily have a one-to-one mapping (Logothetis, 2008). The enhanced frontal response to the oddball task is consistent with previously observed regional functional specialisation and the improved task performance. Brodmann Area 8 is believed to positively correlate with management of uncertainty and expectation, while BA 9 and 10 are part of the dorsolateral prefrontal cortex (DLPFC) responsible for motor planning, organisation, decision making and regulation (Nelson and Luciana, 2001). Cingulate cortex is associated with attention, motivation and error detection. The enhanced frontal response to the oddball task is in strong agreement with the study of Koppelstaetter et al. (2008) who observed an enhancing effect of caffeine on task related BOLD response in brain areas associated with executive functions in a working memory task, albeit with a lower caffeine dose in moderate consumers.

The shortened latency of the oddball ERP in the present study suggests a neuroexcitatory action of caffeine on brain areas involved in executive and attentional functions, consistent with the observed behavioural results. A previous simultaneous EEG–FMRI study of oddball event-related potentials (P300) (Bénar et al., 2007) demonstrated a negative correlation between P300 latency and task-related BOLD signal in the medial frontal cortex. This was revealed in a single-trial analysis exploiting trial to trial variability in response latency. In that study, P300 latency also correlated with reaction times which led to an interpretation of medial frontal cortex being positively related to task performance. The study of Bénar et al. (2007), therefore, supports the notion that our observed alteration of the ERP latency is consistent with the enhanced task-related frontal BOLD response in the present study. However, further investigation would be needed to link directly the alterations in EEG and FMRI responses as the enhanced frontal BOLD response could also be explained by alterations in vascular behaviour, the effects of caffeine being a combination of both neural and vascular (Dunwiddie and Masino, 2001; Haas and Selbach, 2000). Similarly, we cannot rule out a potential neuroexcitatory effect in visual areas but with a different regional effect on the BOLD response, as discussed, due to underlying physiological differences between sensory (e.g. visual) and frontal regions.

### Caffeine and neurotransmitter systems

Caffeine has not only a direct effect on adenosine receptors but also has important secondary effect on other neurotransmitter systems such as cholinergic, noradrenergic and dopaminergic (Ferre, 2008; Latini and Pedata, 2001). A substantial body of evidence indicates that the prefrontal cortex is involved in processes of cognition and receives ascending input from different neuromodulatory systems including dopaminergic (Nelson and Luciana, 2001). As a result of the inhibition of adenosine A_2A_ receptors by caffeine, transmission via dopamine D_2_ receptors is increased (Ferre, 2008; Fredholm et al., 1999). Together with the indication that caffeine affects the attentional system, dopamine D_2_ receptors have been demonstrated to modulate neural networks involved in selective and involuntary attention (Kahkonen et al., 2002). Dopaminergic neurotransmission may play an important role in the generation of the P300. This ERP component is sensitive to dopamine-enhancing drugs in patients with Parkinson's disease (Stanzione et al., 1991; Starkstein et al., 1989). In addition, monoaminergic neurotransmitters were found to supress spontaneous background activity while enhancing cortical neural responses to a stimulus and focussing neural activity to brain structures specific for the processing of particular information (Mattay et al., 2000). This is in agreement with our results as we did not observe significant differences in amplitude or latency of EPs evoked by nontarget or novel stimuli.

Another neurotransmiter, acetylcholine, may attract attention as a target of action for caffeine (Ferre, 2008; Fredholm et al., 1999). For example, Rainnie et al. (1994) demonstrated that caffeine increased firing rates in mesopontine cholinergic neurons, which have been found to participate in the production of arousal evident in EEG. These cholinergic neurons are inhibited by adenosine, providing a coupling mechanism linking EEG arousal and caffeine. This is strong evidence for the role of caffeine in the behavioural state of arousal.

### Methodological considerations

The EEG has the advantage of providing us with an electrophysiological measure which is thought not to be influenced directly by the state of the vasculature. We chose to perform EEG and FMRI simultaneously, whereas, it would have been possible to conduct them in separate sessions and to avoid the potential compromise in quality of simultaneously acquired EEG and FMRI data. For a pharmacological study such as this, the advantage of simultaneous acquisition lies in the guarantee that the volunteer is in the same state for each recording modality. Recording of EEG and FMRI in separate sessions risks adding additional variance to the data because of the potential modulation of brain responses through the environmental influence, e.g. acoustic scanner noise, as well as variation in pharmacological responses which may vary from day to day as a result of differential drug absorption and metabolism.

The 2-way repeated measures ANOVA reveals the influence of caffeine through the interaction effect of the two main factors “dosing” (pre/post) and “drug” (placebo/caffeine). Examination of the regional task-related BOLD signal changes (Fig. S1) suggests a partial contributor to the detected interaction effects may be an increase in BOLD response for visual and motor tasks or a decrease for the oddball task in the DP (post-placebo) condition compared to the BP (pre-placebo) condition. Whilst natural variance in the data could be a source of such apparent differences there may be a ‘time’ effect in which the second scan of the scan day tends to differ from the first. The second scan of the day may be affected by the subject's expectation of a drug effect, or altered alertness or engagement with the task, these effects then being superimposed on the effect of caffeine. We would expect such a time effect to be appropriately modelled by our 2-way analysis in which the effect of caffeine is then isolated by the interaction of the main factors.

We observed caffeine-induced alterations in physiological parameters in our volunteers which have the potential to alter BOLD responsiveness. Caffeine acts as a respiratory stimulant (D'Urzo et al., 1990) causing a significant reduction in end-tidal carbon dioxide. This may contribute to the observed reduction in cerebral perfusion as the brain's blood flow is sensitive to arterial carbon dioxide levels. Brown et al. (2003) have shown that such a reduction in perfusion can give rise to an increase in BOLD contrast. However, while the direction of this change is consistent with BOLD alterations we saw in frontal regions, it is not consistent with the reductions in BOLD contrast in visual and motor cortex. Caffeine also caused a significant increase in systolic blood pressure. The effects of a change in baseline blood pressure on BOLD responsiveness have not been extensively investigated in humans. In rats, transient hypertension has been shown to increase BOLD signal additively with the BOLD response to a simultaneously applied stimulus (Qiao et al., 2007). The rise in blood pressure did not cause a larger CBF response to forepaw stimulation. This observation combined with an expectation that autoregulation of cerebral blood flow may minimise effects of slowly changing blood pressure, suggests that the small changes in blood pressure seen in the current study are unlikely to alter task-related BOLD signal responsiveness.

Caffeine has the potential to alter the dynamics of the haemodynamic response. It has been shown to produce an earlier BOLD response to visual stimulation in the visual cortex (Liu et al., 2004). Examination of the time-course of the BOLD haemodynamic response in the present study suggested that for the visual and motor tasks the principal caffeine effect was of a reduction in the BOLD response amplitude. We were unable to detect a significant caffeine-related change in the time to 50% of the maximum BOLD response amplitude as a marker of the timing of haemodynamic response, although our sampling of the BOLD response was not optimised for this purpose. A small change in timing of the haemodynamic response is unlikely to have a large effect on our detection of changes in task related BOLD activity using a canonical haemodynamic response function (HRF) for the visual and motor tasks because of the block experimental design used. However, our detection of caffeine-related changes to the event-related auditory oddball experiment with a canonical HRF may be more sensitive to caffeine-induced changes in the HRF. Related timing differences could be one source of our observed caffeine effect in the BOLD signal rather than a pure modulation of BOLD response amplitude. That is, if the model and the HRF were temporally shifted with respect to each other, the fitted model amplitude would be altered. Unfortunately given the small signal changes from the auditory oddball task and the rapid event-related design with overlapping HRFs, it was not possible to reliably characterise the HRF within frontal cortex to investigate alterations in HRF timing. We suggest that the previously observed caffeine-induced 1 s temporal shift of the HRF (Liu et al., 2004) is unlikely to give rise to a false activation in this study given that the HRF extends over approximately 10 s and that we have accounted for small shifts in timing by including temporal derivatives in the model. Furthermore, we also suggest that our observed frontal cortical increases in BOLD signal with caffeine on the oddball task are likely to arise principally from a change of BOLD response amplitude because of their agreement with the caffeine-induced signal changes observed by Koppelstaetter et al. (2008) who used a block-design working memory paradigm, this being less sensitive to HRF alterations than our event-related oddball experiment.

We note several limitations of our study. Firstly, we examined only infrequent caffeine consumers. There was no comparison with frequent caffeine consumers which restricts our conclusions to effects of caffeine on naïve subjects. This choice was made to avoid potential confounds associated with frequent caffeine consumption (e.g., (Rogers et al., 2010)) including withdrawal effects or effects of recent dosing on top of the experimental dose. Caffeine versus placebo is generally found to have more marked and somewhat different effects on task performance, mental alertness and mood in consumers (Rogers and Smith, 2011; Rogers et al., 2010). Secondly, a fixed dose of caffeine, not altered for body mass, was administered to participants. This may have increased the variability of caffeine plasma concentrations achieved (which were not measured). This dose effect is likely to be small as we were not able to detect any significant correlations between bodyweight and caffeine induced changes in blood pressure, heart rate, auditory target ERP latency, CBF and BOLD signal. Although we conducted a motor task and were able to measure the FMRI responses to this we were not able to compare them with consistent EEG motor responses, partly due to small head movements inducing artefacts in the EEG at the time of finger tapping.

## Conclusion

In conclusion, our study showed improved performance on an auditory oddball task. We further demonstrated using simultaneous EEG–FMRI differential FMRI effects in infrequent caffeine consumers of a single dose of caffeine on a cognitively engaging task, the auditory oddball, compared with low-level visual and motor tasks. We suggest that this differential effect is likely to reflect a combination of the regional neural influence of caffeine on the cognitive task and regionally dependent caffeine effects on the vascular response. The use of low-level control tasks for comparison with a more cognitively demanding task together with the combined use of EEG and FMRI may help us to distinguish neural from vascular effects of caffeine. This methodology is therefore promising for other pharmacological or disease studies in which the coupling between neural activity and the vascular response may be altered.

The following are the supplementary data related to this article.

Supplementary Tables

Fig. S1 Group mean percentage task-related BOLD signal changes within regions of interest significantly modulated by caffeine. a) Responses to the visual task averaged over the regions of visual cortex and superior parietal lobule shown in Fig. 2b. b) Responses to the motor task averaged over the regions of left sensorimotor cortex shown in Fig. 3b. b) Responses to the auditory oddball task (target–non-target) averaged over the regions of superior frontal gyrus, frontal pole and paracingulate gyrus shown in Fig. 4b. Note that no additional statistical tests were performed on these data as the regions from which they were drawn had already been shown to demonstrate a caffeine effect in the voxel-wise analysis. The errorbars plotted for each session represent the standard deviation for that session. Where sessions are contrasted (DC–DP and BC–BP) the errorbars represent the within-subjects variation, namely the standard deviation of the difference between the sessions. These plotted signal changes are extracted from the linear model fits, namely, a fitted block design for the visual and motor stimuli and contrast of short events for the auditory oddball task using the Featquery tool within FSL.
Abbreviations: baseline placebo (BP), baseline caffeine (BC), placebo (DP), caffeine (DC). Mean and standard deviation across subjects is represented on the bar graphs.

Fig. S2 Time-course representation of the BOLD responses to visual and motor tasks within regions significantly modulated by caffeine. a) Group average responses to the visual task averaged over the regions of visual cortex and superior parietal lobule shown in Fig. 2b. b) Group average responses to the motor task averaged over the regions of left sensorimotor cortex shown in Fig. 3b. Please note that the time-course representations are normalised (scaled) such that the first time point is represented as unit signal. This is to facilitate comparison of the shapes of the BOLD signal responses.
Abbreviations: baseline placebo (BP), baseline caffeine (BC), placebo (DP), caffeine (DC).

Fig. S3 Time-course representation of the BOLD responses to visual and motor tasks within regions significantly modulated by caffeine. a) Group average responses to the visual task averaged over the regions of visual cortex and superior parietal lobule shown in Fig. 2b. b) Group average responses to the motor task averaged over the regions of left sensorimotor cortex shown in Fig. 3b. Please note that the time-course representations are not normalised. They are displayed as raw BOLD signal (arbitrary units).
Abbreviations: baseline placebo (BP), baseline caffeine (BC), placebo (DP), caffeine (DC).

Fig. S4 Group average time-course representation of the BOLD signal response to the auditory oddball task (target stimuli) within the region significantly modulated by caffeine (frontal cortical region shown in Fig. 4b). For convenience of display and to facilitate comparison of relative signal changes the time-course representations are normalised such that the mean value across the time window is unit signal. The oddball task was presented as an event related design in which haemodynamic responses of the different stimulus types overlap (reduced data plotted). Although the stimulus related signal change is small, typically less than 0.1% (and largely negative going, see Fig. S1c) in this frontal region, the full BOLD signal model revealed a statistically significant caffeine effect on the response to target vs. nontarget stimuli for a group-level within-subjects analysis (Fig. 4b).
Abbreviations: baseline placebo (BP), baseline caffeine (BC), placebo (DP), caffeine (DC).

## Figures and Tables

**Table 1:** Physiological parameters measured during caffeine, placebo and baseline conditions (mean ± standard deviation across subjects).

Conditions	Systolic blood pressure (mm Hg)	Diastolic blood pressure (mm Hg)	Pulse Rate (beats per minute)	End-tidal carbon dioxide (mm Hg)^$^
Baseline placebo (BP)	116 ± 9	63 ± 9	85 ± 10	42.0 ± 13.0
Baseline caffeine (BC)	115 ± 9	65 ± 9	87 ± 9	41.7 ± 4.6
(BC-BP)	− 1 ± 10	2 ± 10	2 ± 13	− 0.3 ± 11.3
Placebo (DP)	116 ± 8	65 ± 9	88 ± 6	42.4 ± 11.0
Caffeine (DC)	122 ± 11^+^^⁎§^	70 ± 10^⁎§^	93 ± 8^⁎§^	38.3 ± 4.1^+^^⁎^
(DC–DP)	5 ± 7	5 ± 8	6 ± 8	− 4.1 ± 10.3

^$^Results based on 9 out of 14 subjects.

^+^Significant interaction effect of dosing (baseline or post-dose scan) × drug (placebo or caffeine) in a 2-way repeated-measures ANOVA, p < 0.05.

^⁎^Significantly different with respect to baseline caffeine (two-tailed post-hoc paired *t*-test p < 0.05).

^§^Significantly different with respect to placebo (two-tailed post-hoc paired *t*-test p < 0.05).

**Table 2:** Behavioural performance during the auditory oddball task in caffeine, placebo and baseline conditions (mean ± standard deviation across subjects).

Conditions	Reaction time (ms)	Number of misses	Number of false alarms
Baseline placebo (BP)	603 ± 138	4.7 ± 4.1	1.8 ± 1.5
Baseline caffeine (BC)	609 ± 126	4.9 ± 3.8	1.7 ± 1.6
(BC-BP)	6 ± 59	0.1 ± 1.6	− 0.1 ± 2.0
Placebo (DP)	599 ± 130	5.4 ± 5.2	2.1 ± 1.4
Caffeine (DC)	598 ± 145	2.7 ± 1.9^+⁎§^	1.5 ± 1.2
(DC–DP)	1 ± 81	− 2.7 ± 3.0	− 0.6 ± 1.5

^+^Significant interaction effect of dosing (baseline or post-dose scan) × drug (placebo or caffeine) in a 2-way repeated-measures ANOVA, p < 0.05.

^⁎^Significantly different with respect to baseline caffeine (two-tailed post-hoc paired *t*-test p < 0.05).

^§^Significantly different with respect to placebo (two-tailed post-hoc paired *t*-test p < 0.05).

**Table 3:** Visual evoked potential (VEP) latency and amplitude variations across conditions and electrodes (O1, Oz, O2). There was no significant effect of caffeine on VEP amplitude or latency. (mean ± standard deviation across subjects).

Condition	Latency (ms)			Amplitude (μV)		
O1	Oz	O2	O1	Oz	O2
Baseline placebo (BP)	122 ± 15	120 ± 14	122 ± 14	4.8 ± 2.5	6.1 ± 2.3	5.5 ± 2.5
Baseline caffeine (BC)	118 ± 14	118 ± 15	120 ± 15	5.7 ± 2.4	6.7 ± 2.8	7.0 ± 3.5
(BC–BP)	− 4 ± 11	− 2 ± 8	− 2 ± 7	0.8 ± 2.1	0.6 ± 2.1	1.5 ± 2.5
Placebo (DP)	121 ± 17	116 ± 11	118 ± 13	4.9 ± 2.7	5.4 ± 2.8	5.3 ± 2.8
Caffeine (DC)	117 ± 16	114 ± 13	119 ± 14	5.4 ± 2.3	6.5 ± 3.1	6.4 ± 2.7
(DC–DP)	− 4 ± 14	− 1 ± 13	1 ± 12	0.5 ± 1.6	1.2 ± 2.1	1.2 ± 2.1

**Table 4:** The evoked potential responses for target stimuli in the auditory oddball task: latency and amplitude variations across conditions and electrodes (Fz, Cz, Pz). (mean ± standard deviation across subjects).

Condition	Latency (ms)			Amplitude (μV)		
Fz	Cz	Pz	Fz	Cz	Pz
Baseline placebo (BP)	496 ± 85	508 ± 96	521 ± 90	8.4 ± 3.4	8.6 ± 3.7	10.0 ± 4.2
Baseline caffeine (BC)	524 ± 89	532 ± 87	539 ± 86	8.6 ± 4.0	8.8 ± 3.9	10.2 ± 4.4
(BC–BP)	28 ± 69	24 ± 69	17 ± 102	0.2 ± 1.7	0.2 ± 2.2	0.2 ± 2.9
Placebo (DP)	518 ± 90	523 ± 92	525 ± 98	7.9 ± 3.0	8.1 ± 3.8	10.5 ± 3.3
Caffeine (DC)	424 ± 79^+⁎§^	437 ± 81^+⁎§^	442 ± 83^+⁎§^	8.1 ± 3.6	8.4 ± 3.3	10.1 ± 3.3
(DC–DP)	− 94 ± 38	− 87 ± 36	− 83 ± 46	0.2 ± 2.0	0.3 ± 2.7	− 0.4 ± 1.9

^+^Significant interaction effect of dosing (baseline or post-dose scan) × drug (placebo or caffeine) in a 2-way repeated-measures ANOVA, p < 0.05.

^⁎^Significantly different with respect to baseline-caffeine (two-tailed post-hoc paired *t*-test p < 0.05).

^§^Significantly different with respect to placebo (two-tailed post-hoc paired *t*-test p < 0.05).
