# Supplementary material for: Separating neural and vascular effects of caffeine using simultaneous EEG–FMRI: Differential effects of caffeine on cognitive and sensorimotor brain responses
Source: Neuroimage. 2012 Aug 1;62(1):239–49. doi: 10.1016/j.neuroimage.2012.04.041 (PMC3778750; doi:10.1016/j.neuroimage.2012.04.041)
Supplement: Supplementary Tables [file mmc1.doc]

**SUPPLEMENTARY MATERIAL**

**Table 1S**

Group level BOLD fMRI measured activity for the auditory oddball task (baseline placebo and baseline caffeine scans combined.

Condition: Target *vs.* Non-target

| Region | MNI co-ordinate of peak Z-score within region | Peak Z-score |
| --- | --- | --- |
| L middle frontal gyrus | -28 -20 46 | 4.40 |
| R middle frontal gyrus | 42 38 24 | 5.70 |
| L inferior frontal gyrus | -34 26 4 | 6.18 |
| R inferior frontal gyrus | 38 24 2 | 6.30 |
| Medial frontal gyrus | 0 -8 55 | 5.59 |
| Anterior cingulate gyrus | 0 22 28 | 6.70 |
| L insula | -34 18 4 | 7.56 |
| R insula | 36 24 4 | 6.40 |
| L precentral gyrus | -38 -14 60 | 5.36 |
| R precentral gyrus | 38 -2 65 | 3.56 |
| L postcentral gyrus | -52 -28 50 | 6.53 |
| R postcentral gyrus | 54 -36 50 | 6.67 |
| L superior parietal lobule | -38 -46 54 | 5.16 |
| R superior parietal lobule | 48 -46 54 | 4.17 |
| L supramarginal gyrus | -49 -41 57 | 6.06 |
| R supramarginal gyrus | 58 -38 42 | 6.31 |
| C posterior cingulate gyrus | 0 -28 22 | 6.55 |
| C precuneus | 0 -54 56 | 3.72 |
| L superior temporal gyrus | -50 2 2 | 6.99 |
| R superior temporal gyrus | 52 4 8 | 5.06 |
| L middle temporal gyrus | -53 0 -8 | 6.02 |
| R middle temporal gyrus | 52 -23-10 | 5.7 |
| L inferior temporal gyrus | -42 -14 -16 | 4.07 |
| R inferior temporal gyrus | 42 -18 -16 | 3.01 |
| L parahippocampal gyrus | -24 -2 -14 | 4.85 |
| R parahippocampal gyrus | 24 -4 -14 | 3.65 |
| L lingual gyrus | -2 -92 -4 | 4.18 |
| R lingual gyrus | 2 -70 -2 | 4.85 |
| L thalamus | -18 -22 8 | 4.76 |
| R thalamus | 8 -18 12 | 4.52 |
| L putamen | -26 4 0 | 4.46 |
| R putamen | 32 0 0 | 3.15 |
| L cerebellum | -24-64-26 | 7.22 |
| R cerebellum | 24 -54 -26 | 7.06 |

**Table 2S**

Group level BOLD fMRI measured activity for the auditory oddball task (baseline placebo and baseline caffeine scans combined.

Condition: Target *vs*. Novel

| Region | MNI co-ordinate of peak Z-score within region | Peak Z-score |
| --- | --- | --- |
| R superior frontal gyrus | 28 46 16 | 4.16 |
| R superior/middle frontal gyrus | 34 44 26 | 4.81 |
| L middle frontal gyrus | -34 44 26 | 3.97 |
| R middle frontal gyrus | 42 40 24 | 3.6 |
| C medial frontal gyrus | 0 4 50 | 5.4 |
| C anterior cingulate gyrus | 0 18 26 | 6.36 |
| L anterior cingulate gyrus | -1 20 28 | 6.23 |
| R anterior cingulate gyrus | 2 20 32 | 6.23 |
| L insula | -42 10 -4 | 6.53 |
| R insula | 44 10 -6 | 6.49 |
| L precentral gyrus | -48 -2 8 | 4.92 |
| L postcentral gyrus | -52 -28 46 | 7.43 |
| R postcentral gyrus | 62 -20 22 | 5.74 |
| L superior/inferior parietal lobule | -46 -42 58 | 6.05 |
| R superior/inferior parietal lobule | 48 -32 42 | 4.44 |
| L inferior parietal/supramarginal gyrus | -60 -46 28 | 3.94 |
| R inferior parietal/supramarginal gyrus | 58 -39 38 | 3.58 |
| L precuneus | -3 -66 62 | 3.92 |
| R precuneus | 10 -72 52 | 3.55 |
| L superior temporal gyrus | -46 12 -8 | 5.85 |
| R superior temporal gyrus | 46 12 -8 | 5.66 |
| L amygdala | -24 -2 -16 | 4.72 |
| R amygdala | 22 -2 -16 | 4.72 |
| L putamen/globus palidus | -26 0 -6 | 3.93 |
| R putamen/globus pallidus | 24 -4 -6 | 3.43 |
| L thalamus | -14 -20 5 | 3.77 |
| R thalamus | 14 -12 10 | 3.62 |
| L cerebellum | -24 -56 -26 | 5.02 |
| R cerebellum | 24 -56 -26 | 7.25 |

**Table 3S**

Group level BOLD fMRI measured activity for the auditory oddball task (baseline placebo and baseline caffeine scans combined.

Condition: Novel vs. Non-target

| Region | MNI co-ordinate of peak  Z-score within region | Peak Z-score |
| --- | --- | --- |
| L middle/inferior frontal gyrus | -40 14 16 | 3.69 |
| R middle/inferior frontal gyrus | 38 6 34 | 5.41 |
| L inferior frontal gyrus/insula | -40 18 10 | 4.22 |
| R inferior frontal gyrus/insula | 44 10 24 | 4.54 |
| L posterior cingulate gyrus | -4 -34 22 | 4.86 |
| R posterior cingulate gyrus | 4 -32 22 | 4.72 |
| L planum polare/ superior temporal gyrus | -48 -10 -6 | 5.51 |
| R planum polare/ superior temporal gyrus | 48 -8 -10 | 7.18 |
| L superior temporal gyrus | -64 -24 2 | 5.91 |
| L superior temporal gyrus | -56 -32 6 | 5.50 |
| R superior temporal gyrus | 64 -26 8 | 8.64 |
| R superior temporal gyrus | 58 -28 -6 | 4.37 |
| L middle temporal gyrus | 59 -43 6 | 6.15 |
| R middle temporal gyrus | -59 -44 8 | 4.41 |

**Table 4S**

The evoked potential responses for novel and non-target stimuli of the auditory oddball task: latency and amplitude variations across conditions and electrodes (Fz, Cz, Pz). The evoked potential responses for the main condition if interest, the target stimuli, are given in table 4 in the main text. There were no significant caffeine effects on the novel or non-target evoked potential amplitudes and latencies.

| Condition |  | Novel |  |  |  | Non-target |  |
| --- | --- | --- | --- | --- | --- | --- | --- |
|  | **Fz** | **Cz** | **Pz** |  | **Fz** | **Cz** | **Pz** |
|  | Lat (ms) | Lat (ms) | Lat (ms) |  | Lat (ms) | Lat (ms) | Lat (ms) |
|  | Amp (µV) | Amp (µV) | Amp (µV) |  | Amp (µV) | Amp (µV) | Amp (µV) |
| Baseline Placebo (BP) | 338±65  4.6±2.5 | 346±59  4.3±3.1 | 349±61  3.8±2.0 |  | 300±52  2.5±1.4 | 303±55  2.9±1.8 | 306±46  2.1±1.1 |
| Baseline Caffeine (BC) | 334±70  4.3±2.2 | 341±73  3.7±3.0 | 346±64  3.5±1.6 |  | 298±46  3.4±1.3 | 301±47  3.8±1.8 | 304±42  3.1±1.5 |
| Placebo (DP) | 322±69  4.6±2.1 | 328±74  4.4±2.6 | 335±72  4.1±1.9 |  | 290±48  2.6±1.4 | 294±54  2.8±1.0 | 296±51  2.4±1.3 |
| Caffeine (DC) | 320±62  5.2±2.5 | 324±66  4.3±2.9 | 332±65  4.0±2.8 |  | 279±50  3.3±2.0 | 284±56  3.5±2.2 | 286±53  2.8±1.6 |

**Table 5S**

Cerebral blood flow (ml/100g/min) measured during caffeine, placebo and baseline conditions (mean ± standard deviation across subjects) in gray matter regions of interest defined globally for grey matter and from regions of interest defined functionally by the significant caffeine effects on the BOLD responses in the visual, the motor (finger tapping) and the auditory “oddball” task.

|  | Gray matter | Visual task (visual cortex, and SPL) | Motor task  (left sensorimotor cortex) | Auditory “oddball” task (SFG, frontal pole, PCG) |
| --- | --- | --- | --- | --- |
| Baseline Placebo (BP) | 49.2±9.3 | 46.3±14.2 | 33.3±11.1 | 66.2±15.0 |
| Baseline Caffeine (BC) | 48.8±8.5 | 44.4±11.5 | 33.2±10.2 | 69.9±18.3 |
| (BC-BP) | -0.4±5.3 | -2.0±11.9 | 0.0±9.4 | 3.7±13.8 |
| Placebo (DP) | 50.7±7.5 | 45.9±9.5 | 36.0±8.2 | 71.4±11.9 |
| Caffeine (DC) | 39.5±7.4 ^+^*§ | 31.0±11.7 *§ | 21.8±12.2 *§ | 53.2±13.2^+^*§ |
| (DC-DP) | -11.1±6.3 | -14.5±9.1 | -14.1±10.8 | -18.1±12.4 |

^+^ Significant interaction effect of dosing (baseline or post-dose scan) x drug (placebo or caffeine) in a 2-way repeated-measures ANOVA, p<0.001.

* Significantly different with respect to baseline caffeine (two-tailed post-hoc paired t-test p<0.001)

§ Significantly different with respect to placebo (two-tailed post-hoc paired t-test p<0.001)

Abbreviations: superior parietal lobule (SPL), superior frontal gyrus (SFG) and paracingulate gyrus (PCG).

**Supplementary Figures**

**Fig. S1.** Group mean percentage task-related BOLD signal changes within regions of interest significantly modulated by caffeine. a) Responses to the visual task averaged over the regions of visual cortex and superior parietal lobule shown in Fig. 2b. b) Responses to the motor task averaged over the regions of left sensorimotor cortex shown in Fig. 3b. b) Responses to the auditory oddball task (target – non-target) averaged over the regions of superior frontal gyrus, frontal pole and paracingulate gyrus shown in Fig. 4b. Note that no additional statistical tests were performed on these data as the regions from which they were drawn had already been shown to demonstrate a caffeine effect in the voxel-wise analysis. The errorbars plotted for each session represent the standard deviation for that session. Where sessions are contrasted (DC-DP and BC-BP) the errorbars represent the within-subjects variation, namely the standard deviation of the difference between the sessions. These plotted signal changes are extracted from the linear model fits, namely, a fitted block design for the visual and motor stimuli and contrast of short events for the auditory oddball task using the Featquery tool within FSL.

Abbreviations: baseline placebo (BP), baseline caffeine (BC), placebo (DP), caffeine (DC). Mean and standard deviation across subjects is represented on the bar graphs.

**Fig. S2.** Time-course representation of the BOLD responses to visual and motor tasks within regions significantly modulated by caffeine. a) Group average responses to the visual task averaged over the regions of visual cortex and superior parietal lobule shown in Fig. 2b. b) Group average responses to the motor task averaged over the regions of left sensorimotor cortex shown in Fig. 3b. Please note that the time-course representations are normalised (scaled) such that the first time point is represented as unit signal. This is to facilitate comparison of the shapes of the BOLD signal responses.

Abbreviations: baseline placebo (BP), baseline caffeine (BC), placebo (DP), caffeine (DC).

**Fig. S3.** Time-course representation of the BOLD responses to visual and motor tasks within regions significantly modulated by caffeine. a) Group average responses to the visual task averaged over the regions of visual cortex and superior parietal lobule shown in Fig. 2b. b) Group average responses to the motor task averaged over the regions of left sensorimotor cortex shown in Fig. 3b. Please note that the time-course representations are not normalized. They are displayed as raw BOLD signal (arbitrary units).

Abbreviations: baseline placebo (BP), baseline caffeine (BC), placebo (DP), caffeine (DC).

**Fig. S4.** Group average time-course representation of the BOLD signal response to the auditory oddball task (target stimuli) within the region significantly modulated by caffeine (frontal cortical region shown in Fig. 4b). For convenience of display and to facilitate comparison of relative signal changes the time-course representations are normalized such that the mean value across the time window is unit signal. The oddball task was presented as an event related design in which haemodynamic responses of the different stimulus types overlap (reduced data plotted). Although the stimulus related signal change is small, typically less than 0.1% (and largely negative going, see Fig. S1c) in this frontal region, the full BOLD signal model revealed a statistically significant caffeine effect on the response to target vs. nontarget stimuli for a group-level within-subjects analysis (Fig 4b).

Abbreviations: baseline placebo (BP), baseline caffeine (BC), placebo (DP), caffeine (DC).
