# Supplementary figures and images for: Separating neural and vascular effects of caffeine using simultaneous EEG–FMRI: Differential effects of caffeine on cognitive and sensorimotor brain responses
Source: Neuroimage. 2012 Aug 1;62(1):239–49. doi: 10.1016/j.neuroimage.2012.04.041 (PMC3778750; doi:10.1016/j.neuroimage.2012.04.041)

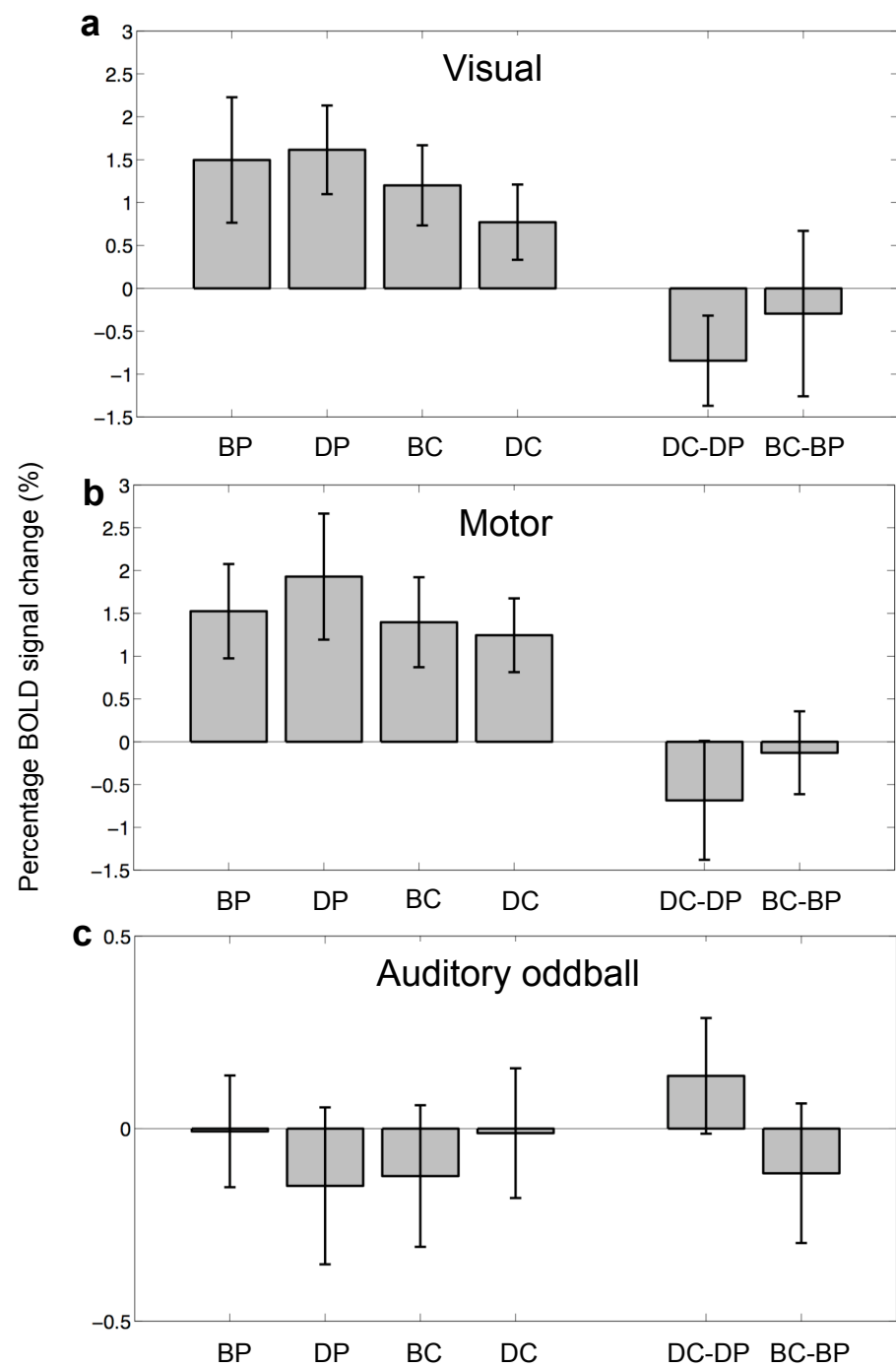

Supplement: Fig. S1 — Group mean percentage task-related BOLD signal changes within regions of interest significantly modulated by caffeine. a) Responses to the visual task averaged over the regions of visual cortex and superior parietal lobule shown in Fig. 2b. b) Responses to the motor task averaged over the regions of left sensorimotor cortex shown in Fig. 3b. b) Responses to the auditory oddball task (target–non-target) averaged over the regions of superior frontal gyrus, frontal pole and paracingulate gyrus shown in Fig. 4b. Note that no additional statistical tests were performed on these data as the regions from which they were drawn had already been shown to demonstrate a caffeine effect in the voxel-wise analysis. The errorbars plotted for each session represent the standard deviation for that session. Where sessions are contrasted (DC–DP and BC–BP) the errorbars represent the within-subjects variation, namely the standard deviation of the difference between the sessions. These plotted signal changes are extracted from the linear model fits, namely, a fitted block design for the visual and motor stimuli and contrast of short events for the auditory oddball task using the Featquery tool within FSL. Abbreviations: baseline placebo (BP), baseline caffeine (BC), placebo (DP), caffeine (DC). Mean and standard deviation across subjects is represented on the bar graphs. [file mmc2.pdf]

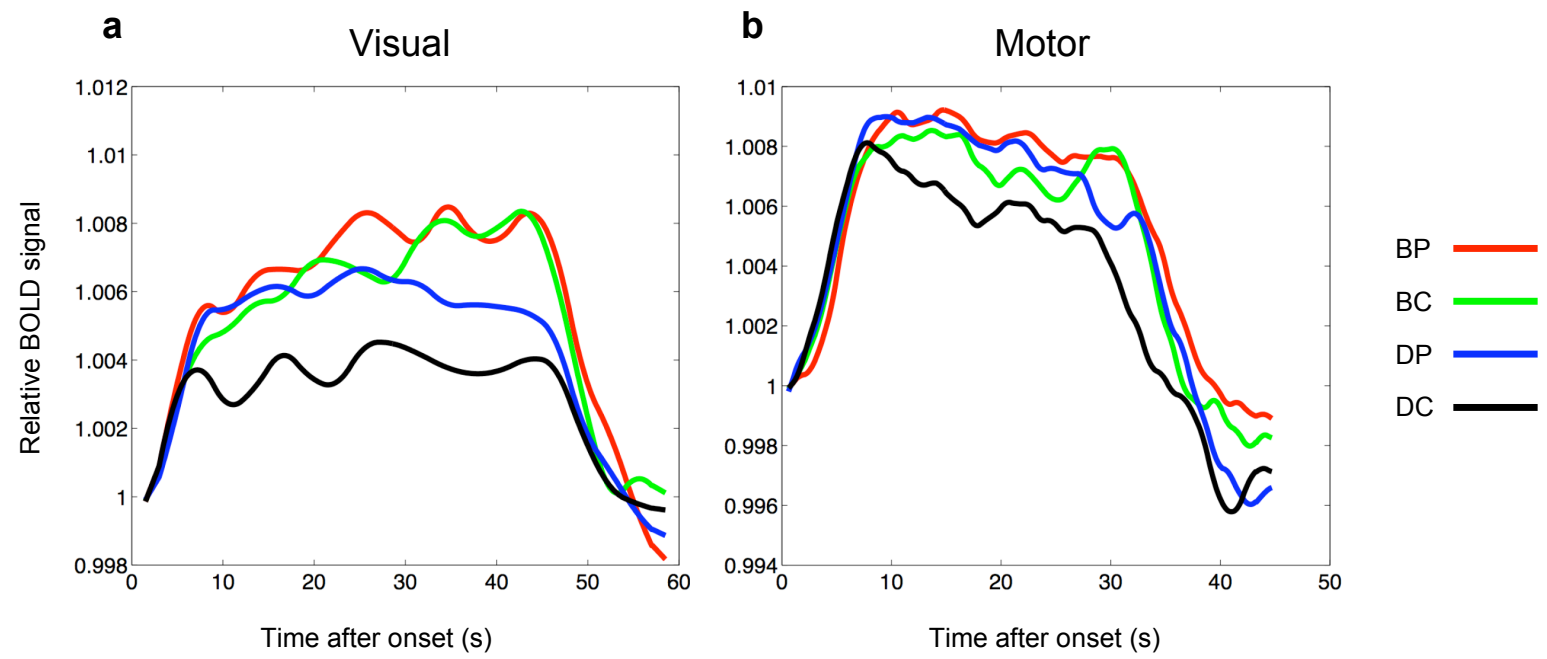

Supplement: Fig. S2 — Time-course representation of the BOLD responses to visual and motor tasks within regions significantly modulated by caffeine. a) Group average responses to the visual task averaged over the regions of visual cortex and superior parietal lobule shown in Fig. 2b. b) Group average responses to the motor task averaged over the regions of left sensorimotor cortex shown in Fig. 3b. Please note that the time-course representations are normalised (scaled) such that the first time point is represented as unit signal. This is to facilitate comparison of the shapes of the BOLD signal responses. Abbreviations: baseline placebo (BP), baseline caffeine (BC), placebo (DP), caffeine (DC). [file mmc3.pdf]

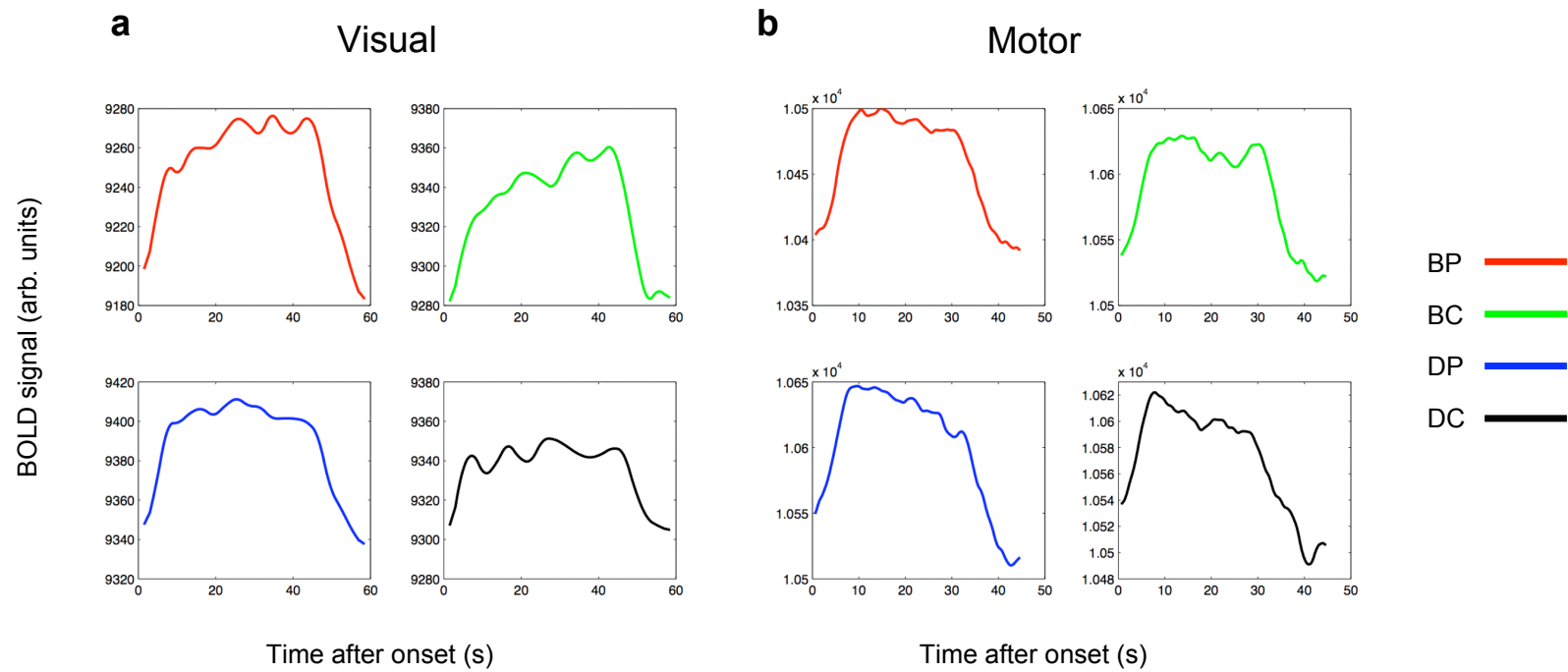

Supplement: Fig. S3 — Time-course representation of the BOLD responses to visual and motor tasks within regions significantly modulated by caffeine. a) Group average responses to the visual task averaged over the regions of visual cortex and superior parietal lobule shown in Fig. 2b. b) Group average responses to the motor task averaged over the regions of left sensorimotor cortex shown in Fig. 3b. Please note that the time-course representations are not normalised. They are displayed as raw BOLD signal (arbitrary units). Abbreviations: baseline placebo (BP), baseline caffeine (BC), placebo (DP), caffeine (DC). [file mmc4.pdf]

### Auditory oddball

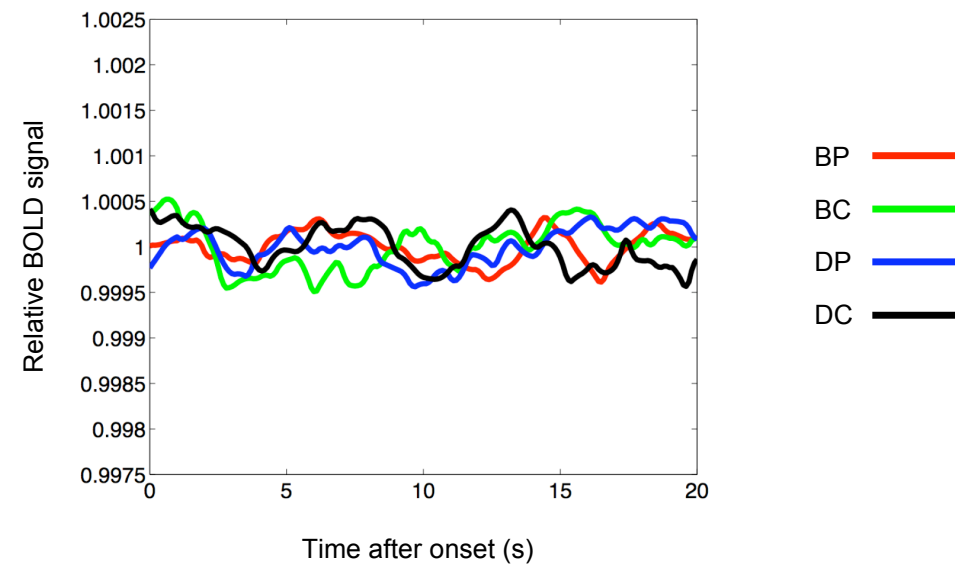

Supplement: Fig. S4 — Group average time-course representation of the BOLD signal response to the auditory oddball task (target stimuli) within the region significantly modulated by caffeine (frontal cortical region shown in Fig. 4b). For convenience of display and to facilitate comparison of relative signal changes the time-course representations are normalised such that the mean value across the time window is unit signal. The oddball task was presented as an event related design in which haemodynamic responses of the different stimulus types overlap (reduced data plotted). Although the stimulus related signal change is small, typically less than 0.1% (and largely negative going, see Fig. S1c) in this frontal region, the full BOLD signal model revealed a statistically significant caffeine effect on the response to target vs. nontarget stimuli for a group-level within-subjects analysis (Fig. 4b). Abbreviations: baseline placebo (BP), baseline caffeine (BC), placebo (DP), caffeine (DC). [file mmc5.pdf]
